# TNF-induced IL-8 and MCP-1 production in the eosinophilic cell line, EOL-1

**DOI:** 10.1155/S0962935196000312

**Published:** 1996-06

**Authors:** L. A. Goldstein, R. M. Strieter, H. L. Evanoff, S. L. Kunkel, N. W. Lukacs

**Affiliations:** 1 Department of Pathology Division of Pulmonary and Critical Care University of Michigan Medical School 1301 Catherine Ann Arbor MI 48109-0602 USA; 2 Department of Internal Medicine Division of Pulmonary and Critical Care University of Michigan Medical School 1301 Catherine Ann Arbor MI 48109- 0602 USA

## Abstract

The role of eosinophils in inflammation and their mode of activation is not well understood. Eosinophil accumulation and subsequent expression of cytokines at the site of inflammation may play a role in exacerbation of inflammatory responses. In the present study, we have examined the role of TNF-α in eosinophil activation and chemokine production using a human leukaemic eosinophil cell line, EOL-1. Initial studies demonstrated that TNF-α induced the upregulation of IL-8 and MCP-1 mRNA and protein. Kinetic studies indicated production of chemokines, IL-8 and MCP-1, as early as 4 h post-activation, with peak levels of chemokine produced at 8 h, and decreasing by 24 h post-TNF-α activation. When IL-10, a suppressive cytokine, was incubated with TNF-α and EOL-1 cells, no effect was observed on IL-8 and MCP-1 production. However, dexamethasone, a glucocorticoid, demonstrated potent inhibitory effects on the EOL-1-derived chemokines. These studies indicate that eosinophils may be a significant source of chemokines capable of participating in, and maintaining, leukocyte recruitment during inflammatory responses, such as asthma.


Mediators of Inflammation, 5, 218–223 (1996)

### TNF-induced IL-8 and MCP-1 production in the eosinophilic cell line, EOL-1

L. A. Goldstein,[1] R. M. Strieter,[2] H. L. Evanoff,[1] S. L. Kunkel[1] and N. W. Lukacs[1,CA]

[1]Department of Pathology and [2] Department of Internal Medicine, Division of Pulmonary and Critical Care, University of Michigan Medical School, 1301 Catherine, Ann Arbor, MI 48109-0602, USA

[CA]Corresponding Author

**The role of eosinophils in inflammation and their mode of activation is not well understood. Eosinophil accumulation and subsequent expression of cytokines at the site of inflammation may play a role in exacerbation of inflammatory responses. In the present study, we have examined the role of TNF-α in eosinophil activation and chemokine production using a human leukaemic eosinophil cell line, EOL-1. Initial studies demonstrated that TNF-α induced the upregulation of IL-8 and MCP-1 mRNA and protein. Kinetic studies indicated production of chemokines, IL-8 and MCP-1, as early as 4 h post-activation, with peak levels of chemokine produced at 8 h, and decreasing by 24 h post-TNF-α activation. When IL-10, a suppressive cytokine, was incubated with TNF-α and EOL-1 cells, no effect was observed on IL-8 and MCP-1 production. However, dexamethasone, a glucocorticoid, demonstrated potent inhibitory effects on the EOL-1-derived chemokines. These studies indicate that eosinophils may be a significant source of chemokines capable of participating in, and maintaining, leukocyte recruitment during inflammatory responses, such as asthma.**

**Key words:**

### Introduction

Eosinophils are granule-containing cells that differentiate from stem cell precursors in the bone marrow, circulate in the blood, and then enter into peripheral tissues.[1,2] Normally present at low concentrations, increased levels of circulating and/or tissue eosinophils have been associated with diseases such as asthma and parasitic infections.[2,3] The presence of eosinophils during chronic inflammatory diseases has been associated with tissue pathology. In addition, eosinophils represent a potential source of cytokines that may influence chronic inflammatory reactions and other biological responses.[1] The establishment of a human eosinophilic leukaemia cell line, EOL-1, has proved to be a useful *in vitro* model for studying the properties of eosinophils and the pathways of their inflammatory responses and released products.

Cytokines regulate immunologic and physiologic events by transmitting information to target cells through receptor–ligand interactions.[4] For example, it has been shown previously that interleukin-1 (IL-1) and tumour necrosis factor-α (TNF-α) act as early response cytokines and can stimulate a variety of both immune and non-immune cells to produce additional peptide mediators, some of which have been shown to be important leukocyte chemotactic factors.[4–6] These chemotactic cytokines, or chemokines, which recruit additional cells to the inflammatory site, can be divided into two polypeptide groups based on the position of two internal disulfide bonds formed from four cysteine amino acid residues. In one supergene family of chemokines (C–C chemokine family), exemplified by the protein monocyte chemoattractant protein-1 (MCP-1), two of the four cysteine amino acid residues are found in juxtaposition to each other. The members of this family are predominantly chemotactic for mononuclear cells, monocytes, and lymphocytes. In the other division of this polypeptide family (C-X-C chemotactic cytokines) two of the four cysteines are separated by one different amino acid.[6,7] This group, typified by interleukin-8 (IL-8), is predominantly chemotactic for neutrophils. Both MCP-1 and IL-8 attract leukocytes to sites of inflammation and have been identified in various diseases such as rheumatoid arthritis, atherosclerosis, as well as in multiple pulmonary diseases.[7,8] In addition, eosinophils have recently demonstrated the ability to produce chemokines.[9,10]

In the present study, we determined the activation pathway of chemokine production of an eosinophilic leukemia cell line, EOL-1. Utilizing this eosinophilic leukaemic cell line we demon-

© 1996 Rapid Science Publishers strated that tumour necrosis factor-α (TNF-α) induces the production of chemokines interleukin-8 and monocyte chemoattractant protein-1. Interestingly, the production of these chemokines was not regulated by a suppressive cytokine, interleukin-10, but could be regulated nonspecifically by dexamethasone. Overall, these studies suggest that eosinophils may contribute to chronic inflammatory responses via their ability to produce chemotactic cytokines, thus amplifying the leukocyte recruitment.

### Materials and Methods

*Cell culture:* The eosinophilic cell line EOL-1 was maintained in RPMI 1640 medium supplemented with 2 mM L-glutamine, 24 mM Hepes, 100 μg/ml penicillin, 100 μg/ml streptocycin (Hazelton Research Products) and 10% heat-inactivated foetal calf serum (FCS) (Gibco Laboratories). The isolation and characterization of EOL-1 have been previously described.[11]

*Assessment of cytokine levels by specific ELISAs:* Specific eosinophil-derived cytokines were quantified using a modification of a double ligand ELISA method as previously described.[12] Briefly, flat-bottomed 96-well microtitre plates were coated with 50 μl/well of rabbit antichemokine (IL-8 and MCP-1) antibodies (1 μg/ml in 0.6 mol/l NaCl, 0.26 ml/l $H_3BO_4$, and 0.08 N NaOH, pH 9.6, for 16h at 4°C and then washed with BPS (pH 7.5) 0.05% Tween-20 (wash buffer). Nonspecific binding sites on microtitre plates were blocked with 2% BSA in PBS and incubated for 90 min at 37°C. Plates were rinsed three times with wash buffer and diluted eosinophil-derived conditioned media (50 μl) was added, followed by incubation for 1h at 37°C. Plates were washed three times with wash buffer, 50 μl/well of biotinylated rabbit anticytokine (IL-8 and MCP-1) antibodies were added for 45 min at 37°C. After washing, 100 μl peroxidase conjugated Avidin (Dako Corp, 1:5000 dilution) was added and incubated 30 min at 37°C. Plates were again washed three times and then chromogen substrate OPD was added. The plates were incubated at room temperature to the desired extinction, the reaction terminated with 50 μl/well of 3 M $H_3SO_4$ solution and samples were read at 490 nm in an ELISA reader. The sensitivity of this ELISA method is consistently greater than 50 pg/ml.

*Northern blot analysis of chemokine mRNA:* Total RNA from the EOL-1 cell line was isolated, as previously described.[12,13] Briefly, a solution containing 25 mM Tris, pH 8.0, 4.2 M guanidine isothiocyanate, 0.5% Sarkosyl, and 0.1 M 2-mercaptoethanol was overlaid on eosinophil monolayers. After homogenization, the above suspension was added to a solution containing an equal volume of 100 nM Tris, pH 8.0, 10 mM EDTA, and 1.0% SDS. Then, the mixture was extracted using chloroform–phenol (1:1: v/v) and chloroform–isoamyl alcohol (24:1 v/v). The total RNA was precipitated by using alcohol and the pellet dissolved in diethyl pyrocarbonate (DEPC)-treated $H_2O$. Total RNA was separated by electrophoresis using denated formaldehyde, 1% agarose gels, followed by transblotting to nitrocellulose. Blots were baked, prehybridized, and hybridized with a P-5′ end-labelled oligonucleotide probe specific for IL-8 or MCP-1. The 30-mer oligonucleotide probes for IL-8 and MCP were complementary to the nucleotide sequences 5′-GTT-GGC-GCA-GTG-TGG-TCC-ACT-CTC-AAT-CAC-3′ and 5′-TTG-GGT-TTG-CTT-GTC-CAG-GTG-GTC-CAT-GGA-3′, respectively. Blots were stringently washed after hybridization and exposed to X-ray film. Specific chemokine mRNA was quantified using imaging analysis video densitometry with a frame grabber (Image Capture 1000; Scion Corp., Walkersville, MD) and Image 1.49 software (National Institutes of Health Public Software, Bethesda, MD).

*Statistical analysis:* Data were analysed by Macintosh II computer using a statistical software package (Statview II; Abacus Concept, Inc., Berkeley, CA) and expressed as mean ± S.E.M. Data that appeared statistically significant were compared by Student's *t*-test for comparing the means of multiple groups and considered significant if $p$ values were $<0.05$.

### Results

*Cytokine activation of EOL-1 cells:* Previously, it has been shown that eosinophils produce cytokines, such as TNF-α, which can act on a variety of cell types to upregulate further cytokine production.[14] In the present study, we assessed the production of chemokines, IL-8 and MCP-1, from the eosinophilic cell line, EOL-1. Various concentrations, 0.1, 1.0, and 10 ng/ml, of IL-1, IFN-γ, IL-4, IL-10, and TNF-α were incubated with the EOL-1 cells ($1 \times 10^6$/ml). After 24h, supernatants were collected and analysed for IL-8 and MCP-1 by specific ELISAs. Only TNF-α-activated EOL-1 cells showed significant cytokine production. None of the other cytokines examined induced chemokine production from the EOL-1 cells. Figures 1 and 2 show a dose-dependent increase in IL-8 and MCP-1 production occurring when EOL-1 cells were activated with TNF-α.

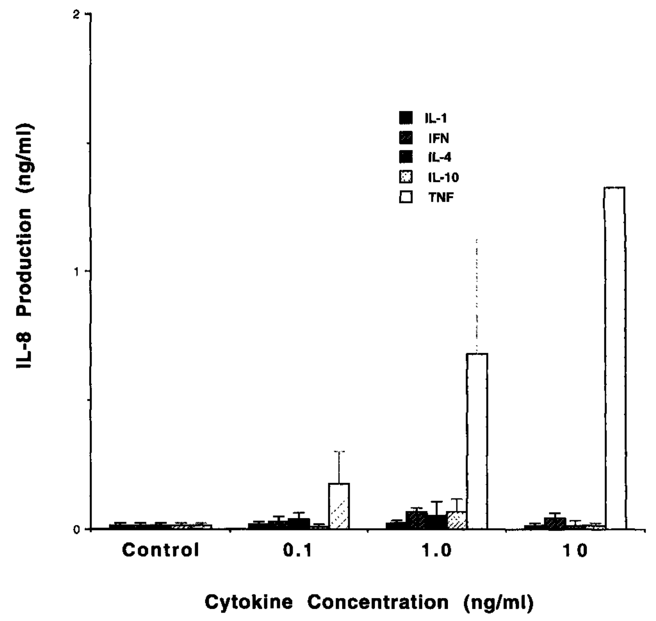

FIG. 1. IL-8 production from cytokine activated EOL-1 cells. Various concentrations (0.1, 1.0, and 10 ng/ml) were used to stimulate EOL-1 cells. Culture supernatants were collected at 24 h and measured by IL-8 specific ELISA. Data represents the mean ± S.E.M. of three repeat experiments.

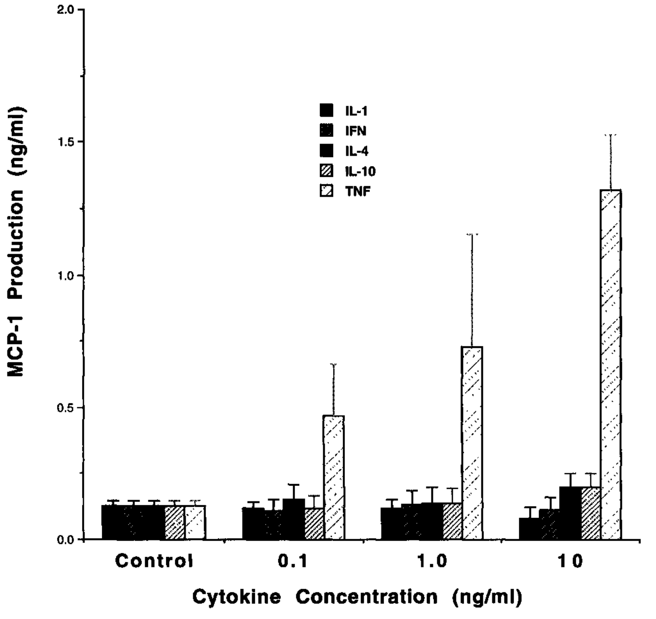

FIG. 2. MCP-1 production from cytokine activated EOL-1 cells. Various concentrations (0.1, 1.0, and 10 ng/ml) were used to stimulate EOL-1 cells. Culture supernatants were collected at 24 h and measured by MCP-1 specific ELISA. Data represents the mean ± S.E.M. of three repeat experiments.

Peak IL-8 and MCP-1 production was observed at a TNF concentration of 10 ng/ml.

*Time-dependent production of EOL-1-derived chemokines in response to TNF:* To determine the temporal production of IL-8 and MCP-1, EOL-1 cells were cultured at 1, 4, 8, or 24 h at 37°C with 10 ng/ml of TNF-α and assessed by specific ELISAs for IL-8 and MCP-1. As depicted in Figs 3 and 4, EOL-1 cells did not release appreciable levels of IL-8 or MCP-1 within the first hour. By 4 h, significant levels of IL-8 and MCP-1 were detected that continued to rise and peak at 8 h. By 24 h post-TNF-α activation cytokine levels had decreased (IL-8) or were maintained (MCP-1). Figure 5 shows representative Northern blots of

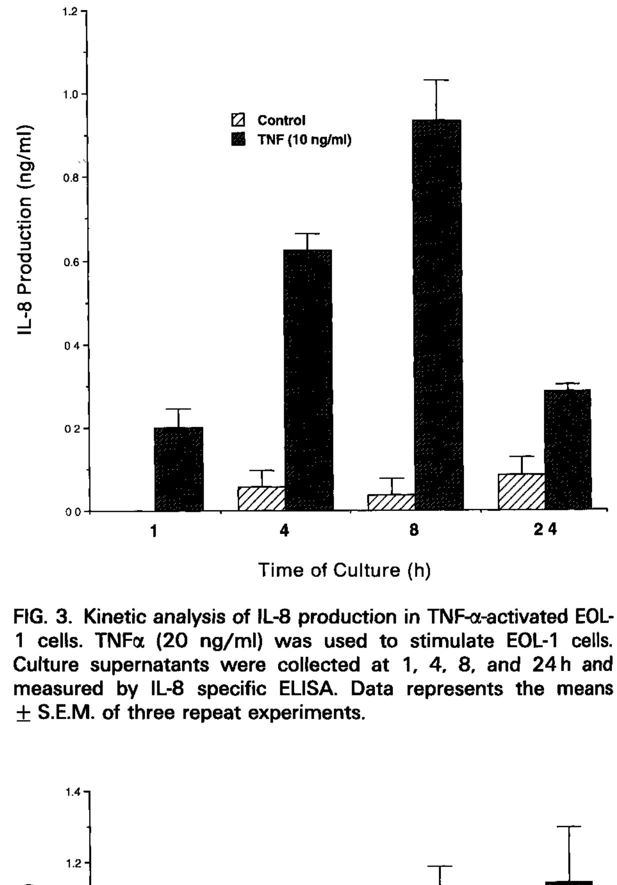

FIG. 3. Kinetic analysis of IL-8 production in TNF-α-activated EOL-1 cells. TNFα (20 ng/ml) was used to stimulate EOL-1 cells. Culture supernatants were collected at 1, 4, 8, and 24 h and measured by IL-8 specific ELISA. Data represents the means ± S.E.M. of three repeat experiments.

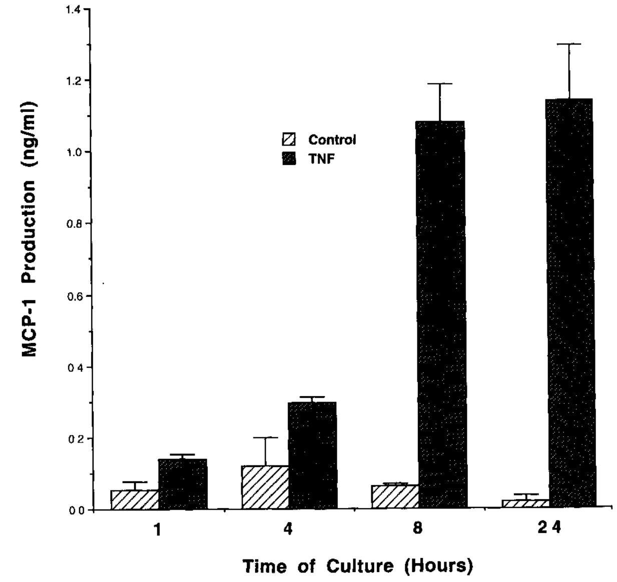

FIG. 4. Kinetic analysis of MCP-1 production in TNF-activated EOL-1 cells. TNF-α (20 ng/ml) was used to stimulate EOL-1 cells. Culture supernatants were collected at 1, 4, 8, and 24 h and measured by MCP-1 specific ELISA. Data represents the means ± S.E.M. of three repeat experiments.

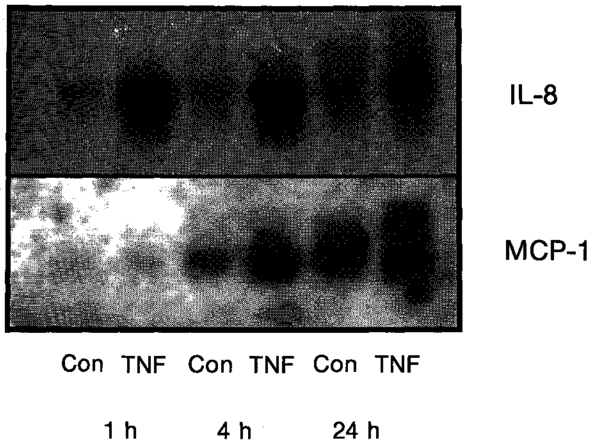

FIG. 5. Representative Northern blots showing kinetic analysis of EOL-1-derived steady state IL-8 and MCP-1 mRNA production. TNF-α (10 ng/ml) was used to stimulate EOL-1 cells. RNA extractions were performed on EOL-1 cells cultured at 1, 4, and 24 h. Repeat experiments showed similar results.

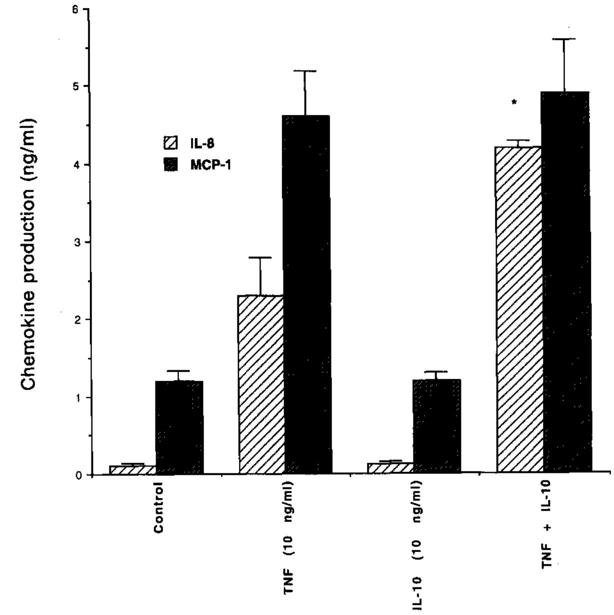

FIG. 6. Effect of IL-10 on IL-8 and MCP-1 production in TNF-activated EOL-1 cells. IL-10 (10 ng/ml) was added to the TNF-activated (10 ng/ml) EOL-1 cells. Culture supernatants were collected at 8 h and assayed for IL-8 and MCP-1 by specific ELISAs. Data represents the means ± S.E.M. of three repeat experiments.

EOL-1-derived steady state chemokine mRNA production. Strong expression of IL-8 mRNA occurs at 1 and 4 h post-TNF-α (10 ng/ml) activation with continued expression at 24 h post-TNF-α. MCP-1 mRNA production is expressed at 4 h post-TNF-α (10 ng/ml) with increased expression at 24 h post-TNF activation. Interestingly, MCP-1 mRNA expression increased in controls at 4 and 24 h timepoints.

*Suppression of EOL-1-derived chemokines:* To determine the regulation of IL-8 and MCP-1, EOL-1 cells were cultured with either IL-10 or dexamethasone. IL-10 is known to inhibit cytokine synthesis in other leukocyte populations,[15–19] whereas dexamethasone, a glucocorticoid, appears to nonspecifically shutdown cellular activation events. TNF-α (10 ng/ml)-stimulated EOL-1 cells were incubated with or without IL-10 (10 ng/ml). Supernatants were collected after 8 h and samples were assessed for cytokine production by specific ELISAs. TNF-α-stimulated EOL-1 cells incubated with IL-10 did not show significant differences in IL-8 or MCP-1 (Fig. 6) production. In fact, IL-10 appeared to augment the TNF driven IL-8 production ($p < 0.05$). Northern blot analysis confirmed these data, demonstrating no decrease in either IL-8 or MCP-1 mRNA expression when IL-10 was added to the cultures (data not shown). In contrast, when EOL-1 cells were challenged with TNF-α in the presence of various doses of dexamethasone ($10^{-7}$ to $10^{-5}$ M), a significant decrease in both IL-8 and MCP-1 production was observed (Fig. 7). Altogether, these data indicate that although EOL-1-derived chemokine production can be inhibited by dexamethasone,

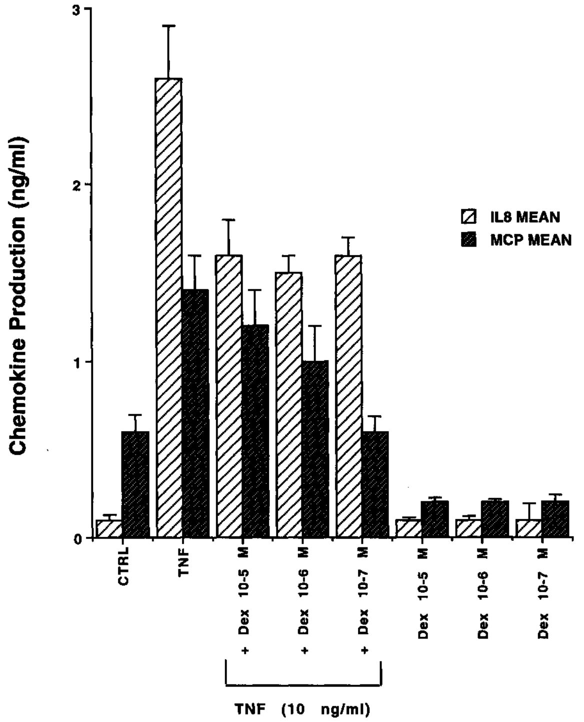

FIG. 7. Effect of dexamethasone on chemokine production in TNF-α-activated EOL-1 cells. Dexamethasone ($10^{-6}$ to $10^{-7}$ M) was added to TNF-α-activated (10 ng/ml) EOL-1 cells. Culture supernatants were collected at 8 h and assayed for IL-8 and MCP-1 by specific ELISAs. Data represents the means ± S.E.M. of three repeat experiments.

it appears that the regulation of these chemokines by IL-10 is different compared with other leukocyte populations.

### Discussion

Eosinophilic infiltrations are found in chronic inflammatory responses, such as those observed during parasitic and allergic inflammation[2,3] and may play a role in exacerbation of inflammatory/allergic reactions by perpetuation of leukocyte recruitment. A better understanding of the eosinophil activation pathway and the subsequent cytokine production cascade would provide useful information for application in immunotherapy during chronic inflammation. Our studies have confirmed the importance of TNF-α as a key activator of eosinophil-like EOL-1 cell induction of IL-8 and MCP-1 mRNA expression and protein production. Dose response studies demonstrated that TNF-α was capable of significant activation of the IL-8 and MCP-1 from EOL-1 cells. Time course studies showed that cytokine production occurred within 4 h of incubation with TNF and that culture of eosinophils with TNF-α for 8 h induced peak levels of IL-8 and MCP-1. Interestingly, incubation of IL-10 with TNF-α and EOL-1 cells did not affect IL-8 and MCP-1 production, while EOL-1 cells could be regulated by dexamethasone. These results suggest that EOL-1 cells are regulated by IL-10 differently than other leukocyte populations. Overall, these studies suggest that eosinophils may significantly contribute to the exacerbation of chronic inflammation and upregulate the leukocyte extravasation through the production of chemokines.

Activation of eosinophils and the subsequent release of IL-8 and MCP-1 may be an important contributing factor during a chronic inflammatory response. In particular, the infiltration of eosinophils into the lung tissue is known to occur during the late phase of asthma.[20–23] It has been previously shown that BAL cells from asthmatics, as compared to normal controls, express increased levels of TNF-specific mRNA.[24] This increased production of TNF, which occurs in conjunction with the presence of eosinophils, may be a contributing factor for activating the eosinophils leading to upregulated chemokine production. Interestingly, recent studies have identified eosinophils as a source of TNF[1] suggesting that they can also contribute to the overall activation of inflammatory responses.

The chemokines, IL-8 and MCP-1, which were found to be produced by the EOL-1 cells, have been associated with a number of inflammatory diseases. IL-8 production has been observed in cystic fibrosis, IPF, emphysema, ARDS, bronchiestasis, and chronic bronchitis.[25] IL-8 levels have also been detected during late-phase reactions of asthma.[26] In addition, IL-8 is known to be a chemoattractant for neutrophils,[27,28] eosinophils,[29] and has been implicated as a lymphocyte chemotactic protein.[10] These findings suggest that eosinophil-derived IL-8 may recruit additional leukocytes into tissue during an inflammatory/allergic reaction, thus exacerbating the overall response.

MCP-1 is a potent monocyte chemoattractant[30] and recently has been found to act as a T-lymphocyte chemoattractant.[10] Activated T-lymphocytes, in addition to eosinophils, have been documented to accumulate in tissue during chronic inflammation and more specifically during late-phase asthmatic reactions.[31] MCP-1 made by eosinophils may augment T-lymphocyte mediated responses via its ability to recruit various leukocyte populations to the site of inflammation. Furthermore, eosinophils found at sites of active fibrosis have been shown to express MCP-1 mRNA[10] suggesting that they may play an important role in chronic fibrosing diseases.

Finally, we determined IL-10 had a minimal role in regulating EOL-1-derived IL-8 and MCP-1. IL-10 is a cytokine produced by CD4+ cells, B cells, certain populations of CD8+ cells, Epstein–Barr Virus (EBF)-transformed lymphoblastoid cell lines, monocytes and macrophages.[25,32–34] In addition, IL-10 demonstrates varied immunosuppressive bioactivity on cytokine production and, along with IL-4 and IL-5, is produced during allergic responses.[35] IL-10 downregulates IFN-γ production associated with Th1 responses as well as monocyte and polymorphonuclear (PMN)-derived cytokine production, including IL-8.[36] In this study we have demonstrated that IL-10 does not inhibit cytokine production from TNF-activated EOL-1 cells. Since eosinophil accumulation appears to be enhanced during Th2 responses and IL-10 does not regulate this response, eosinophils may play a significant role in maintaining chronic allergic and parasitic responses. However, since IL-10 strongly regulates the production of TNF from leukocyte populations IL-10 may indirectly affect the cytokine production *in vivo* from eosinophilic cells. A final possibility may be that the EOL-1 cells do not have IL-10 receptors and therefore cannot respond to the cytokine. Unlike IL-10, dexamethasone did inhibit chemokine production, indicating that the regulation within these cells is similar to other leukocyte populations. The dexamethasone induced inhibition of the chemokines correlates well with the successful use of steroids in treatment of chronic eosinophilic disease states, such as asthma.

Our findings suggest that eosinophils may participate in inflammatory/allergic reactions through the generation of chemokines, IL-8 and MCP-1, further augmenting leukocyte recruitment. In addition, IL-10, an inhibitor of monocyte and PMN-derived cytokines, did not regulate EOL-1-derived cytokine production, suggesting altered regulation of this leukocyte subset.

### References

1. Costa JJ, Matossian K, Resnik MB, *et al.* Human eosinophils can express the cytokines tumor necrosis factor-α and macrophage inflammatory protein 1-α. *J Clin Invest* 1993; **91**: 2673.
2. Wardlaw AJ. Eosinophils in the 1990s: new perspectives on their role in health and disease. *Postgrad Med J* 1994; **70**: 536.
3. Smith H. Asthma, inflammation, eosinophils and bronchial hyperresponsiveness. *Clin Exp Allergy* 1992; **22**: 187.
4. Kunkel SL, Strieter RM. Cytokine networking in lung inflammation. *Hosp Pract* 1990; **25(10)**: 63.
5. Strieter RM, Koch AE, Antony VB, Fick RB, Standiford TJ, Kunkel SL. The immunopathology of chemotactic cytokines: the role of interleukin-8 and monocyte chemoattractant protein-1. *J Lab Clin Med* 1994; **123**: 183.
6. Kunkel SL, Lukacs NW, Strieter RM. The role of interleukin-8 in the infectious process. *Ann NY Acad Sci* 1994; **730**: 135.
7. Strieter RM, Kunkel SL. The immunopathology of chemotactic cytokines. In: Lindley IJD, ed. *The Chemokines.* New York: Plenum Press, 1993.
8. Standiford TJ, Kunkel SL, Basha MA, *et al.* Interleukin-8 gene expression by a pulmonary epithelial cell line. *J Clin Invest* 1990; **86**: 1945.
9. Kita H, Abu-Ghazaleh RI, Sur S, Gleich GJ. Eosinophil major basic protein induces degranulation and IL-8 production by human eosinophils. *J Immunol* 1995; **154**: 4749.
10. Zhang K, Gharaee-Kermani M, Jones ML, Warren JS, Phan SH. Lung monocyte chemoattractant protein-1 gene expression in bleomycin-induced pulmonary fibrosis. *J Immunol* 1994; **153**: 4733.
11. Saito H, Bourinbaiar A, Ginsburg M, *et al.* Establishment and characterization of a new human eosinophilic leukemia cell line. *Blood* 1985; **66(6)**: 1233.
12. Kasama T, Strieter RM, Lukacs NL, Burdick MD, Kunkel SL. Regulation of neutrophil-derived chemokine expression by IL-10. *J Immunol* 1994; **152**: 3559.
13. Streiter RM, Kasahara K, Allen R, *et al.* Cytokine-induced neutrophil-derived interleukin-8. *Am J Pathol* 1992; **141**: 397.
14. Strieter RM, Remick DG, Ham JM, Colletti LM, Lynch III JP, Kunkel SL. Tumor necrosis factor-alpha gene expression human whole blood. *J Leukocyte Biol* 1990; **47**: 366.
15. Bogdan C, Vodovotz Y, Nathan C. Macrophage deactivation by IL-10. *J Exp Med* 1991; **174**: 1549.
16. De Waal Malefyt R, Abrams J, Bennett B, Figdor CG, de Vries JE. Interleukin 10 (IL-10) inhibits cytokine synthesis by human monocytes: an autoregulatory role of IL-10 produced by monocytes. *J Exp Med* 1991; **174**: 1209.
17. Bogdan C, Paik J, Vodovotz Y, Nathan C. Contrasting mechanisms for suppression of macrophage cytokine release by transforming growth factor-β and interleukin-10. *J Biol Chem* 1992; **267**: 23301.
18. Fiorentino DF, Zlotnik A, Mosmann TR, Howard M, O'garra A. IL-10 inhibits cytokine production by activated macrophages. *J Immunol* 1991; **147**: 3815.
19. Ralph P, Nakoinz I, Sampson-Johannes A, Fong S, Lowe D, Min HY, Lin L. IL-10, T lymphocyte inhibitor of human blood cell production of IL-1 and tumor necrosis factor. *J Immunol* 1992; **148**: 808.
20. Durham SR, Kay AB. Eosinophils, bronchial hyperreactivity and late phase asthmatic reaction. *Clin Allergy* 1985; **15**: 411.
21. Metzger WJ, Hunninghake GW, Richerson HD. Late phase asthmatic responses: inquiry to mechanism and significance. *Clin Rev Allergy* 1985; **3(2)**: 145.
22. De Monchy JG, Kauffman HF, Venge P, *et al.* Bronchoalveolar eosinophilia during allergen-induced late phase asthmatic reactions. *Clin Rev Allergy* 1985; **3**: 145.
23. Ohashi Y, Motojima S, Fukuda T, Makino S. Airway hyperresponsiveness, increased intracellular spaces of bronchial epithelium, and increased infiltration of eosinophils and lymphocytes in bronchial mucosa in asthma. *Am Rev Respir Dis* 1992; **145**: 1469.
24. Ying S, Robinson DS, Varney V, *et al.* TNF-alpha mRNA expression in allergic inflammation. *Clin Exp Allergy* 1991: **21(6)**: 745.
25. Shute J. Interleukin-8 is a potent eosinophil chemo-attractant. *Clin Exp Allergy* 1994; **24**: 203.
26. Metzger WJ, Richerson HB, Worden K, Monick M, Hunninghake GW. Bronchoalveolar lavage of allergic asthmatic patients following allergen bronchoprovocation. *Chest* 1986; **89**: 477.
27. Baggiolini M, Walz A, Kunkel SL. Neutrophil-activating peptide-1/interleukin-8, a novel cytokine that activates neutrophils. *J Clin Invest* 1989; **84**: 1045.
28. Leonard EJ, Skeel A, Yoshimura T, Noer K, Kutvirt S, van Epps D. Leukocyte specificity and binding of human neutrophil attractant/activation protein-1. *J Immunol* 1990; **144**: 1323.
29. Warringa RAJ, Mengelers HJJ, Raaijmakers JAM, Bruijnzeel PLB, Koenderman L. Upregulation of formyl-peptide and interleukin-8-induced eosinophil chemotaxis in patients with allergic asthma. *J Allergy Clin Immunol* 1993; **91**: 1198.
30. Carr MW, Roth SJ, Luther E, Rose SS, Springer TA. Monocyte chemoattractant protein-1 acts as a T-lymphocyte chemoattractant. *Proc Natl Acad Sci USA* 1994; **91**: 3652.
31. De Monchy JG, Kauffman HF, Venge P, *et al.* Bronchoalveloar eosinophilia during allergen-induced late phase asthmatic reactions. *Am Rev Respir Dis* 1985; **426**: 373.
32. Larsen CG, Anderson AO, Apella E, Oppenheim JJ, Matushima K. Neutrophil activating protein (NAP-1) is also chemotactic for T-lymphocytes. *Science* 1989; **260**: 355.
33. Taub DD, Conlon K, Lloyd AR, Oppenheim JJ, Kelvin DJ. Preferential migration of activated CD4+ and CD8+ T cells in response to MIP-1a and MIP-1b. *Science* 1993; **260**: 355.
34. Westwick J, Li SW, Camp RD. Novel neutrophil-stimulating peptides. *Immunol Today* 1989; **10**: 146.
35. Smith DR, Kunkel SL, Burdick MD, Wilke CA, Orringer MB, Whyte RI, Strieter RM. Production of interleukin-10 by human bronchogenic carcinoma. *Am J Pathol* 1994; **145**: 18.
36. Kasama T, Strieter RM, Lukacs NW, Burdick MD, Kunkel SL. Regulation of neutrophil-derived chemokine expression by IL-10. *J Immunol* 1994; **152**: 3559.

ACKNOWLEDGEMENTS. This work was supported in part by grants from NIH (AI36302 and HL35276) and the American Lung Association.

**Received 5 March 1996; accepted in revised form 1 April 1996**
